# Dry Electrode Processing Technology and Binders

**DOI:** 10.3390/ma17102349

**Published:** 2024-05-15

**Authors:** Kaiqi Zhang, Dan Li, Xuehan Wang, Jingwan Gao, Huilin Shen, Hao Zhang, Changru Rong, Zheng Chen

**Affiliations:** 1 Key Laboratory of High-Performance Plastics, Ministry of Education, National and Local Joint Engineering Laboratory for Synthesis Technology of High-Performance Polymers, College of Chemistry, Jilin University, Changchun 130012, China; zhangkq22@mails.jlu.edu.cn (K.Z.); xuehan23@mails.jlu.edu.cn (X.W.); shenhl22@mails.jlu.edu.cn (H.S.); haozhang23@mails.jlu.edu.cn (H.Z.); 2 National Key Laboratory of Advanced Vehicle Integration and Control, China FAW Group Co., Ltd., Changchun 130013, China; lidan@faw.com.cn (D.L.); gaojingwan@faw.com.cn (J.G.)

**Keywords:** lithium-ion batteries, dry electrode technology, dry spraying deposition, polymer fibrillation

## Abstract

As a popular energy storage equipment, lithium-ion batteries (LIBs) have many advantages, such as high energy density and long cycle life. At this stage, with the increasing demand for energy storage materials, the industrialization of batteries is facing new challenges such as enhancing efficiency, reducing energy consumption, and improving battery performance. In particular, the challenges mentioned above are particularly critical in advanced next-generation battery manufacturing. For batteries, the electrode processing process plays a crucial role in advancing lithium-ion battery technology and has a significant impact on battery energy density, manufacturing cost, and yield. Dry electrode technology is an emerging technology that has attracted extensive attention from both academia and the manufacturing industry due to its unique advantages and compatibility. This paper provides a detailed introduction to the development status and application examples of various dry electrode technologies. It discusses the latest advancements in commonly used binders for different dry processes and offers insights into future electrode manufacturing.

## 1. Introduction

In recent years, the rapid advances in electric vehicles has led to an increased demand for lithium-ion batteries (LIBs) among consumers. This demand is accompanied by escalating performance expectations, particularly in areas such as storage capacity and production costs [1,2,3,4,5,6,7]. Increased storage capacity has the potential to address the predominant issue of poor battery life in electric vehicles. Additionally, a reduction in the cost of individual batteries could lead to a decrease in the overall selling price of the vehicle, thereby fostering the widespread adoption and advancement of electric vehicles. The inevitable development direction of power batteries is achieving high performance at a low cost [8]. In addition to the development of new high energy density active energy storage materials or new battery structures, the resolution of the aforementioned issues can also be achieved through the optimization and innovation of the lithium battery production process [9,10,11,12,13,14].

At this stage, the predominant method employed by the majority of battery manufacturers for battery electrode production is the conventional slurry-casting (SC) process, also referred to as the wet process [15]. Taking the cathode as an example, the battery production process utilizing this method typically involves the following steps: initially, a slurry based on N-methyl pyrrolidone (NMP) containing an active material (e.g., lithium iron phosphate, etc.), a conductive agent (e.g., conductive carbon black, etc.), a binder (e.g., polyvinylidene fluoride, etc.), and several other additives is applied onto a collector (Al foil or Cu foil). Subsequent procedures such as drying, cutting, and assembly are then conducted. The production process described is firmly established and currently serves as the primary method of production. However, there are still some serious problems with this processing method, such as: (1) the large amount of NMP can be harmful to the human reproductive system and can also seriously pollute the surrounding environment [16,17]; (2) the price of NMP solvent is high, and thus accounts for a high proportion of the cost in the production of electrodes [18,19]; (3) during the solvent drying process, the solvent molecules at the bottom move upward, resulting in the large volume contraction of the electrode structure and cracks, and the poor distribution of components such as binder, thus affecting the performance of the battery [20,21]; (4) the drying and recycling process of solvent is seriously time-consuming and energy-consuming [22,23]. This is a problem not only in the production of cathodes, but also in anodes. Even though water can be used as the solvent of binder for anodes, which can further reduce the cost of solvents, the damage to the human body, and the degree of environmental pollution [24,25], problems (3) and (4) still cannot be solved. In addition, the electrodes produced by the wet process have relatively low energy storage density because the active layer thickness is only 50–200 μm.

Hence, stemming from the first nature principle, the innovative concept of eliminating solvents in electrode processing was introduced, leading to extensive research endeavors on the “solvent-free electrode processing technology” [26], and the technological innovation was realized, which is called the “dry electrode technology”. At this stage, “dry electrode technology” has become a research hotspot in the field of secondary power batteries, and a variety of dry processing technologies have been widely reported, some of which are still in the stage of basic research (e.g., polymer fibrillation), and some of which have begun to be applied to commercial production (e.g., powder compression), which shows great commercial value and practical value.

Previous reviews [27,28,29] have compared the dry processing technologies with the conventional wet processing technology and presented the application of the dry processing technologies in the preparation of electrodes and electrolytes for batteries. In this paper, we provide an overview of emerging dry processing technologies for electrodes, analyze and summarize the process characteristics and binders for each processing method, and finally discuss the challenges and future prospects for the development of this field.

## 2. Dry Processing Methods for Electrodes

According to the distinct process characteristics involved in electrode dry processing technology, the current methods for electrode dry processing are primarily categorized into five types: dry spraying deposition, melt extrusion, 3D printing, powder compression, and polymer fibrillation. While each of the aforementioned methods possesses unique technical characteristics, the overall process remains largely consistent. The process primarily comprises three steps: dry mixing, dry coating (dry deposition), and pressing into the final electrode.

### 2.1. Dry Spraying Deposition

Dry spraying deposition (DSD) is a typical method of dry processing and preparation of electrodes [30,31,32,33]. This method typically involves several essential processing steps. Firstly, the active material, conductive agent, binder, and other crucial components are uniformly dispersed and compounded using a mechanical mixing device to produce powder granules within a specific size range (100 µm to 500 µm). Subsequently, the aforementioned powder particles are evenly deposited onto the metal collector’s surface to create an electrode active layer of a designated thickness with the aid of external methods. The external methods commonly employed include air spraying [31], automatic electrostatic spraying [34,35,36,37], and manual electrostatic spraying [38,39]; of these, the automatic electrostatic spraying and the manual electrostatic spraying are collectively known as “electrostatic spraying”. Finally, the active layer deposited on the collector is further transformed into a thin and dense electrode active layer through direct hot pressing or roller pressing. During this process, the active layer and the metal collector are more tightly bonded together due to the pressure and shear force exerted.

**Figure 1:**
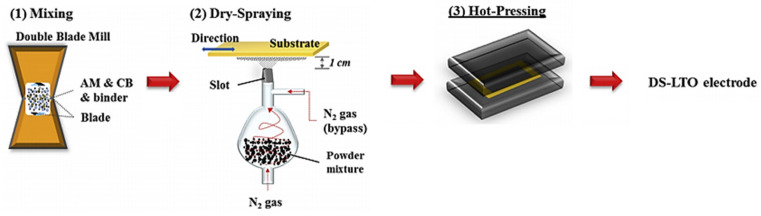
Schematic diagram of lithium titanate electrodes prepared by DSD method [31]: (**1**) mixing of electrode materials by a double-blade mill; (**2**) spraying under N_2_ condition; (**3**) hot pressing into electrodes at 175 °C.

In 2015, Dong-Won Park et al. [31] were the first to document their research on the fabrication of anodes for lithium-ion batteries using the air spraying method, as illustrated in Figure 1. Lithium titanate (Li_4_Ti_5_O_12_) was selected as the active material, carbon black (CB) as the conductive agent, and poly(vinylidene fluoride) (PVDF) as the binder. Initially, the active material, conductive agent, and binder were combined in a double-blade mill at a mass ratio of 8:1:1 for 5 min to prepare a powder mixture suitable for spraying (see Figure 1(1)). The composite powder of the electrode mentioned above was subsequently applied onto the metal substrate (aluminum foil) using dry spraying equipment, with the assistance of a high-pressure N_2_ gas flow. Subsequently, the material was transferred to a hot press machine and subjected to hot pressing at 175 °C (0.59 MPa). The battery performance of the electrode was then evaluated under various hot-pressing durations (30 min, 45 min, 60 min, and 90 min). In the half-cell experiment utilizing lithium foil as the anode, it was observed that the electrode subjected to a hot-pressing duration of 60 min exhibited the most optimal battery performance. Specifically, it demonstrated a capacity retention rate of 69.4% after 100 cycles at a current density of 1C.

**Figure 2:**
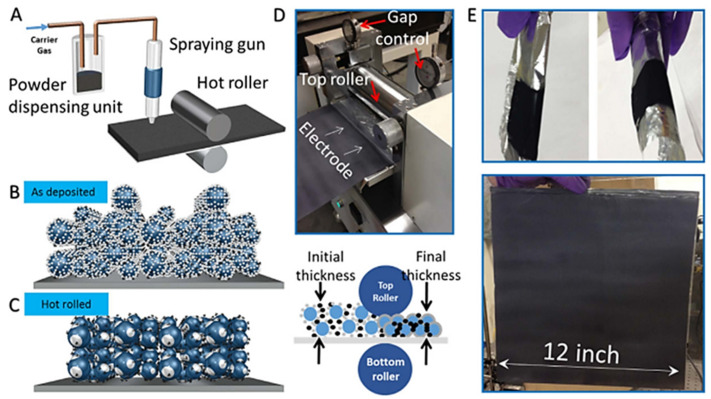
Illustration of a typical electrode manufacturing DSD process [32]. (**A**) Manufacturing system for electrodes created by DSD method. (**B**) 3D representation of a DSD electrode before thermal activation. (**C**) 3D representation of a DSD electrode after hot rolling and thermal activation. (**D**) Hot roller configuration. (**E**) DSD electrodes on Al foils.

In 2016, Ludwig et al. [32] reported a method for preparing electrodes using the electrostatic spraying deposition method. It is obviously different from the air spraying process mentioned above in that the electrostatic spraying method is firstly used instead of the air spraying method to complete the deposition and film-forming operation of the powder mixture on the surface of the metal collector, and then the roller pressing is used instead of the hot pressing to complete the pressing and molding operation of the electrode. The advantage of electrostatic spraying is that it can deposit the powder mixture into the active layer [29] more quickly and conveniently, and roller pressing can better control the thickness and density of the film [40]. As shown in Figure 2, the main process is as follows: firstly, LiCO_2_ (LCO), C65 (Super C65 carbon), and PVDF are mixed with a mass ratio of 90:5:5 to make a powder mixture, followed by using a high-pressure electrostatic spray gun to uniformly deposit the powder on a metal collector (Al foil) ground at one end; the deposited semi-finished electrode was then heat-treated at 250 °C for 1 h (for activated PVDF [41,42]), and then the deposited active layer was tightly pressed with the Al foil using a roll-pressing device to obtain electrodes. The mechanical properties of the dry electrodes and the corresponding SC electrodes were tested and the performance of the batteries (half batteries assembled with lithium foil as counter electrodes) were tested, and the mechanical properties and cycling performance of the dry electrodes were better than those of the corresponding SC electrodes (Table 1).

The uniform distribution of electrode materials on the surface of the collector is the key to the preparation of high-quality dry electrodes. In order to investigate the mixing of electrode materials in the dry powder coating process, they used the same method [32] to prepare a 12-inch square electrode sheet, in which the active material can reach 98% by mass, and the content of conductive additives and binder is as low as 1 wt.%; After assembling a half-cell with the prepared electrode as the cathode and the lithium foil as the anode, the battery performance test was carried out: the capacity of the battery can reach 134 mAh/g at a current density of 0.1C, its capacity can reach 127.8 mAh/g at a current density of 0.5C, and the battery can still maintain 77% of the initial capacity after 100 cycles [30]. Based on this electrode system, the authors further developed a corresponding interfacial energy physical model [33]. The theoretical calculations show that, compared to the SC electrode, the binder and conductive additives of this electrode can still be uniformly distributed on the surface of the LCO particles despite the low content of the conductive additives and binder in the active layer of the electrode. The authors believe that the excellent performance of the half-cell and results of theoretical calculations can prove that the dry spraying method is conducive to achieving a good distribution of the binder and the conductive additive in the active layer of the electrode.

**Figure 3:**
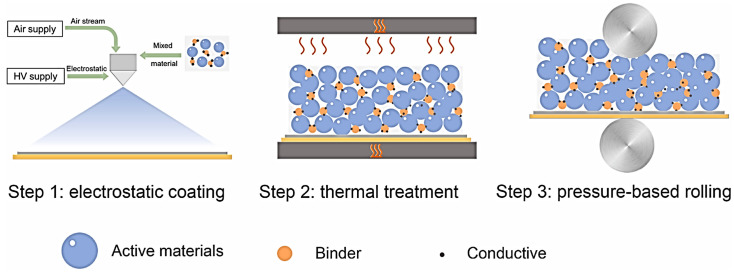
Large-area preparation of NCM anode by DSD method [38].

In 2021, Enmeng Zhen et al. [38] carried out research work on dry electrodes using a manual electrostatic spraying method. As shown in Figure 3, LiNi_0.5_Co_0.2_Mn_0.3_O_2_ (NCM523) was used as the active material, and PVDF and carbon black were used as the binder and conductive additive, respectively. Firstly, different mass ratios of NCM523, PVDF, and CB (90:5:5, 87.5:7.5, and 85:10:5) were mixed in a ball mill at 200 rpm for 1 h to prepare powdered active composite granular materials that could be used for spraying, and then the active powder materials were uniformly sprayed by a Nordson Encore LT manual spray gun onto one end of grounded carbon-coated Al foil. Subsequently, the Al foil deposited with the active material was heat-treated at 200 °C for 2 h and then rolled at room temperature to obtain electrode sheets with 35–40% porosity and 30μm thickness. For comparative research work, SC electrodes with the same electrode material composition and ratio were also prepared. The half-cells were assembled with lithium foil as counter electrodes and battery performance tests were carried out, which showed that the capacity retention rate of the battery based on dry electrodes (69%) was significantly higher than that of the battery based on SC electrodes (52%) after 300 cycles.

**Figure 4:**
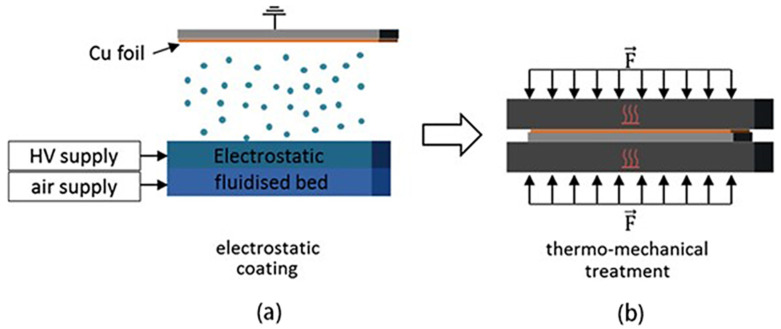
Preparation of graphite anode by electrostatic spraying method: (**a**) electrostatic spraying process; (**b**) hot pressing [34].

The dry spraying deposition process is applicable not only for cathode preparation but also for anode preparation. In 2019, Schälicke et al. prepared a series of graphite anodes employing various binders through the application of fluorine-containing thermoplastic polymers (such as tetrafluoroethylene, hexafluoropropylene, fluorinated ethylene vinylidene (THV), and fluorinated ethylene propylene (FEP)) [34] using electrostatic spraying and hot-pressing techniques. The deposition process was efficiently completed in only 2 s under a high-voltage electric field (10–25 kV) (Figure 4a). The active layer that was deposited underwent additional hot pressing (200 kN, 170–300 °C) for 2–5 min to produce the final electrode (Figure 4b). Subsequently, half-cells were constructed using the electrodes obtained and lithium foils as the counter electrodes for testing. The capacity of these cells reached up to 370 mAh/g (99% of the theoretical capacity), with a capacity retention rate of 97% after 50 cycles at 0.5C. The results indicate that the electrochemical performance is similar to that of the wet process and is anticipated to supplant the traditional SC electrode production technology.

The dry spraying deposition method has better compatibility with common lithium-ion battery active materials (especially inorganic cathode active materials) [27]. The binder can be selected from a wide range of options. The active materials, conductive agent, and binder are evenly distributed [30], and the battery performance is comparable to that of the corresponding wet-processed battery. However, the method still needs to be further improved in terms of active material loading, electrode active layer thickness, and uniformity of electrode component distribution. In addition, the research into the dry spraying deposition method is only carried out on a laboratory scale, and the compatibility of this process with the current stage of commercial production equipment for lithium-ion batteries is not significant, so the electrode production efficiency cannot be compared with that of the SC process in practical applications.

### 2.2. Melt Extrusion

The melt extrusion method typically entails the initial pre-mixing of the active material, conductive agent, and binder, followed by their introduction into a twin-screw extruder. The mixture undergoes shear compounding within the twin-screw to attain the intended dispersion effect. During this process, the binder gradually melts as the temperature increases incrementally, ensuring the uniform and secure adhesion of the active material and the conductive agent. The active mixture, coated with the molten resin binder, is extruded through the die opening and formed into a self-supporting film of the desired thickness using a roll-pressing apparatus. Following this, the self-supporting film is bonded to the metal collector either using roll-pressing or hot-pressing equipment, resulting in the production of the final electrode [43,44]. The thermal stability and mechanical processing characteristics of the binder play a crucial role in the preparation of optimal dry electrodes using the melt-extrusion method. PVDF exhibits favorable thermal stability, enabling it to retain its physical and chemical characteristics over extended periods at elevated temperatures. Consequently, PVDF holds promise for potential application as a binder in the melt extrusion technique [45].

Researchers have implemented the processing strategy of “sacrificing some or all of the binder” to enhance the processing performance and augment the porosity of the electrodes. Polymer binders possessing facile processing characteristics, low thermal degradation thresholds, and environmentally sustainable degradation byproducts have been employed in the process of melt extrusion. The polymer binders fulfill the essential processing criteria for fabricating electrode sheets through melt extrusion and exhibit thermal decomposition properties at elevated temperatures. The decomposition of the binder facilitates the creation of voids and channels within the electrode film, which are conducive to ion transport. Consequently, this process can significantly enhance the porosity of the electrodes. As a result, this type of polymer is commonly referred to as a “sacrificial” binder, with polypropylene (PP) and polypropylene carbonate (PPC) being typical representatives. In addition to “sacrificial” binders, melt extrusion can also use “permanent” binders [46], typically represented by PVDF [47] and rubber materials [48]. Melt extrusion involving sacrificial binders differs from melt extrusion using “permanent” binders. The melt extrusion method utilizing a sacrificial binder involves an additional step of “binder removal” in contrast to the melt extrusion method employing a “permanent” binder. This process can be divided into four main steps: (1) mixing of active materials; (2) melt extrusion and molding of self-supporting electrode film; (3) calendering of electrodes; and (4) binder removal.

In 2019, Sotomayor et al. prepared high energy density electrodes using lithium iron phosphate (LFP) and lithium titanate (LTO) as the positive and negative active materials, respectively, and carbon as the conductive agent [49]. High-energy density electrodes were prepared by melt extrusion using a four-stage process of dry mixing, extrusion, binder removal, and sintering. A mixture of polypropylene (PP, 50 vol%), paraffin (PW, 46 vol%), and stearic acid (SA, 4 vol%) was used as a “sacrificial” binder. The active material and binder were first mixed well, and then the mixture was introduced into a twin-screw extruder and extruded at 160–170 °C. The extrusion process was repeated three times to obtain semi-finished electrodes. For binder removal, PW and SA were first removed at 200 °C, and then the temperature was raised to 450 °C to remove PP; this operation resulted in the creation of pores inside the electrode. Finally, the electrode was subjected to a sintering process at 800 °C for 2 h, which can further enhance the bonding between the remaining particles in the active layer of the electrode, improve the adhesion between the active layer and the Al foil, and stabilize the pore structure. The sintered LTO and LFP electrodes with thicknesses of 550 μm and 500 μm, respectively, were used to assemble a full cell with LFP as the cathode and LTO as the anode, and the specific capacity of the cell area was 13.3 mAh/cm^2^, with a capacity retention rate of 75.7% over 50 cycles at C/12. This research work demonstrates that it is feasible to fabricate high area capacity electrodes by melt extrusion using a sacrificial binder strategy.

**Figure 5:**
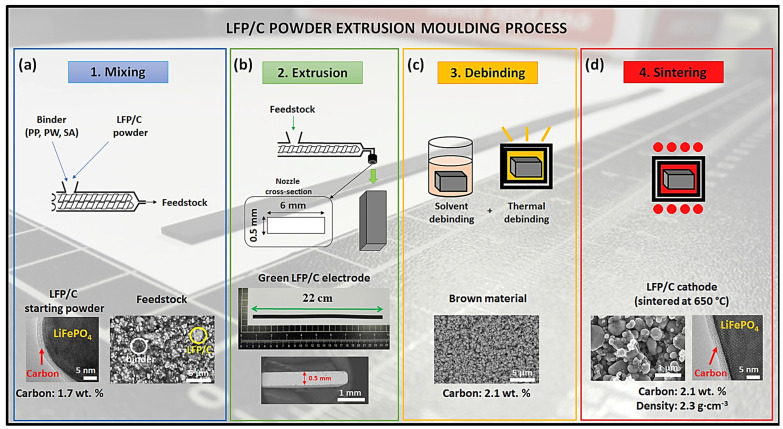
Schematic diagram of the fabrication of LFP electrodes by melt extrusion process using PP, PW, and SA binder system [50]. (**a**) Mixing of electrode materials. (**b**) Extrusion procedure. (**c**) Solvent debinding and thermal debinding procedures. (**d**) Composite electrodes sintered at 650 °C.

Torre-Gamarra et al. [50] carried out further research work on the preparation of high energy density dry electrodes using the same sacrificial binders (PP, PW, and SA). In order to prepare LFP electrodes with a high thickness, they optimized the binder removal process and were able to successfully prepare binder-free self-supported LFP electrodes with a thickness of up to 500 μm (Figure 5). In the binder removal procedure, the binder-containing electrode self-supported active layer film was first impregnated with n-heptane at 50 °C to remove PW and SA, which resulted in the formation of a certain degree of network channel structure in the dense electrode active layer film. These channels facilitate the expulsion of gases generated in the further heating process. PP is next removed by thermal degradation. The thermal degradation process was accomplished by going through two constant temperature platforms heated at 200 °C and 450 °C, respectively, with the former favoring the elimination of undissolved PW and SA and the latter thermally degrading the PP. The half-cells were assembled with lithium foil as counter electrodes, and the half-cells exhibited high area capacity (13.7 mAh/cm^2^) and good cycling stability (capacity retention of around 80% after 40 cycles) at low current densities (C/24–C/10), which provided a new idea for the preparation of lithium-ion batteries with high energy densities using the melt extrusion method.

**Figure 6:**
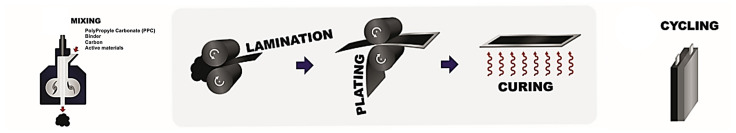
Preparation of LTO, LFP, or NCM electrodes by melt extrusion using PPC as a processing aid [48].

In 2020, Khakani et al. [48] reported the preparation of a series of dry electrodes with different active materials by melt extrusion using a binder system consisting of polypropylene carbonate (PPC) and hydrogenated nitrile rubber (HNBR). The HNBR acts as a permanent binder (Figure 6), while the PPC is used both as a processing aid and as a sacrificial binder, which promotes the mixing of the composite electrode components and will be removed at a later stage of the process, so that the electrodes obtain the desired porosity. Therefore, in this research work, the amount of PPC plays an important role in influencing the change in electrode porosity. Firstly, HNBR and PPC were introduced into an internal mixer at 90 °C to obtain a homogeneous molten blend. Then, active materials (LTO, LFP, or NCM) and conductive additives (C65 + carbon nanofiber) were added to the HNBR/PPC for compound dispersion to produce electrode composites containing binders. The above composite was rolled and adhered to the carbon-coated Al foil several times at 40–60 °C to obtain semi-finished electrodes with thickness (400 μm) and porosity (30%). Finally, LFP, the NCM111 cathode, and LTO anode were successfully prepared by adding and removing PPC at 230 °C with an active material mass fraction of 77.5%. The LFP cathode and LTO anode were selected to assemble the full cell, which exhibited a capacity of 123 mAh/g at a high current density of 5C; the cell had excellent cycling performance (90% capacity retention after 250 cycles). It was shown that the preparation of electrodes using different active materials does not affect the porosity modulation effect of the sacrificial binder on the active layer of the electrode; moreover, the dry electrodes prepared by this method are also comparable to the traditional SC electrodes in terms of electrochemical performances.

In the same year, Astafyeva et al. [46] prepared LiNi_0.8_Co_0.15_Al_0.05_O_2_ (NCA, 90 wt.%) electrodes and graphite electrodes with a high content of active material using PPC as a “sacrificial” binder by the melt extrusion method. As shown [46] in Table 2, the mass ratio of each component in the active layer changed significantly before and after the removal of the “sacrificial” binder, and the ratio of the active material could be increased to more than 90%. The half-cell with a high NCA content cathode and a lithium foil anode exhibited a high specific capacity of 160 mAh/g at 0.5C. In addition, the porosity of the electrode can be effectively regulated by adjusting the amount of PPC.

Melt extrusion represents a scalable method for fabricating electrodes, characterized by a high loading of active materials. It serves as an efficient approach for producing thick electrodes with a high energy density. Nevertheless, the extrusion process is influenced by the condition of the pellets and necessitates the meticulous management of various critical factors, including the extrusion temperature, shear force, and extrusion duration [27]. Furthermore, challenges such as excessive polymer consumption, complex fabrication procedures, and elevated sintering temperatures impede its broader utilization in the practical production of electrodes.

### 2.3. 3D Printing

The 3D printing technology is a rapid prototyping technology, also known as additive manufacturing technology. It is commonly used to manufacture 3D structural products or parts with complex and special structural patterns, and its application scope has been further expanded to the field of battery production in recent years. In electrode manufacturing, two 3D printing technologies, liquid deposition modelling (LDM) [51,52] and fused deposition modelling (FDM) [53] have been successfully applied. Compared to the LDM method, FDM is a solvent-free, dry electrode technology. FDM, also known as fused filament manufacturing [54], is a widely used and easy to process 3D printing technology. FDM employs a specific high-temperature-resistant nozzle that extrudes and deposits an electrode composite containing a polymer binder onto the metal collector at a certain rate at a temperature higher than the polymer’s melting temperature. The nozzle moves horizontally according to a set procedure to create the target electrodes layer by layer. It is easy to see that the process and principle of electrode preparation by 3D printing is very similar to that of fused deposition. However, the advantage of 3D printing technology is that the shape, morphology, and thickness of the electrodes can be precisely tailored to the specific application scenario. Due to the application limitations of the fused deposition method, and in combination with the basic requirements of battery electrodes for polymer binders, the polymer binders that can be used at this stage are mainly based on polymers such as poly(lactic acid) (PLA), poly(polycarbonate) (PC), and acrylonitrile–butadiene–styrene (ABS) [55,56].

**Figure 7:**
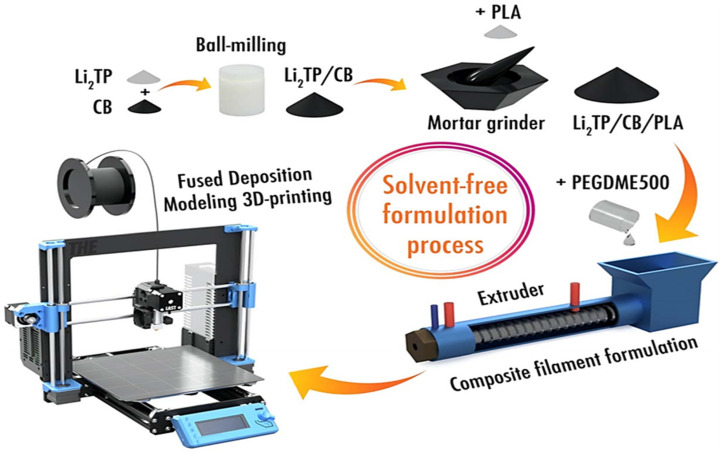
Schematic diagram of solvent-free 3D-printed electrode process [57].

In 2021, Alexis Maurel et al. [57] prepared a more environmentally friendly lithium terephthalate (Li_2_TP)/PLA composite filament for 3D printing by FDM technology to be used as an anode material for lithium-ion batteries (shown in Figure 7). The homemade Li_2_TP and CB were firstly mixed at a mass ratio of 4:1 and ball-milled for 15 min; then, the powder mixture was mixed with PLA powder and added to a Filabot Original extruder; subsequently, the plasticizer PEDGME500 was added to the extruder, and the composite ratio of the mixture in the extruder was Li_2_TP/PLA/CB/PEDGME500 = 4:4:1:1 wt.%. The composite filaments (1.75 mm in diameter) were obtained by the extruder at 175 °C and can be used for 3D printing. Finally, round electrode sheets of 12.7 mm diameter, 200 μm or 600 μm thick, were obtained by printing on Cu foil using a Prusa MK3S 3D printer with the composite filament as raw material. After assembling the half-cell with lithium foil as the counter electrode, it was tested and exhibited a discharge capacity of 237 mAh/g at low current density (C/40) for the first cycle. This study provides a feasible reference for the fabrication of next-generation 3D-printed lithium-ion batteries using FDM technology.

**Figure 8:**
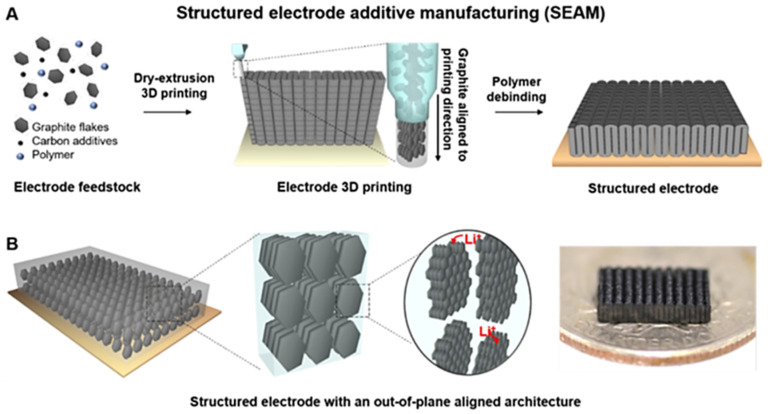
Schematic of additive manufacturing of 3D graphite thick electrode structure with out-of-plane aligned structure [58]: (**A**) schematic diagram of graphite electrodes prepared by 3D printing; (**B**) schematic and photograph of the multi-scale out-of-plane aligned 3D graphite electrode.

In 2022, Soyeon Park et al. [58] prepared a blend using graphite as the active material, carbon black, and multi-walled carbon nanotubes (CNTs) as the conductive additives, and PLA as the sacrificial binder, and melted and extruded the blend into a composite filament that could be used for 3D printing using a single-screw extruder. Then, the electrode active layer was printed on the Cu foil in accordance with a pre-set printing procedure, obtaining an electrode sheet containing the PLA binder. Next, the above electrode sheet was heat-treated at 600 °C to remove the PLA, thereby obtaining a freestanding 3D graphite electrode made of graphite and carbon additives (mass ratio of 7:3) (Figure 8). A half-cell assembled with lithium foil as the counter electrode was tested, and the capacity retention was 86.4% after 50 cycles at 0.1C. Compared with the SC-processed cell, the specific capacity and area capacity at 1C were increased by 200% and 260%, respectively, which has significant potential to be further applied to the practical production of high-capacity electrodes.

Using 3D printing technology, it is possible to precisely customize the scale dimensions of electrodes to specific application scenarios [59]. However, there are some issues with 3D printing technology, namely that the process is not suitable for large-scale electrode production manufacturing, but only for specific scenarios, such as microelectronics and wearable devices [60]. Furthermore, the 3D printing process is slightly more demanding in terms of equipment requirements compared to several other processes.

### 2.4. Powder Compression

The powder compression method uses a compressible active material to prepare the active layer of the electrode. Compared with other dry electrode technologies, the most obvious difference in the processing of the powder compression method is that while other dry electrode technologies involve dry coating or dry deposition, the powder compression method omits this process. The powder compression method is a simple process: the dry battery material powder is mixed and pressed directly into the electrode by means of a hot press or a hydraulic press, so the powder compression method is also known as the direct pressing method.

**Figure 9:**
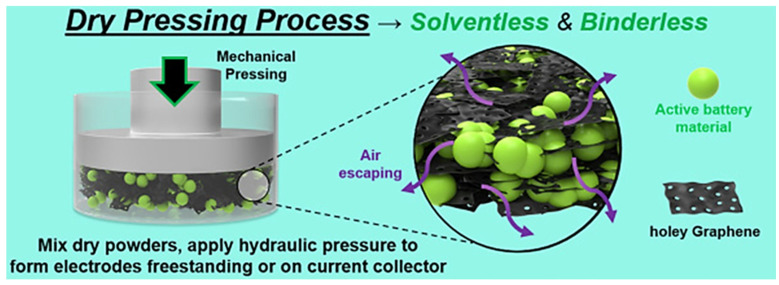
Preparation of LFP anode by direct pressing using compressible porous graphene [61].

The powder compression method can be performed without adding binder. In 2019, Kirsch, D. J et al. mixed different active materials such as NCM, LFP, LCO, and porous graphene and then pressed them directly with a hydraulic press (20–500 Mpa) to form electrodes (Figure 9). Among them, the LFP cathode showed a rate performance and cycle performance (capacity retention > 90% after 100 cycles at 0.2C) in a half-cell [61]. The porous graphene is the key material for the process because the presence of voids within the faces allows for the easy transfer of matter; porous graphene has been shown to be a superior electrode material to ordinary graphene for application in the powder compression method. This porous graphene powder can also be easily pressed into different shapes of dense and strong electrodes without any solvent or binder at room temperature, and it has high electrical conductivity and other properties that are important for the electrochemical performance; therefore, it has great potential to be applied in the production of electrodes for lithium-ion batteries [62]. 2020, Brandon A. Walker et al. [63] combined porous graphene with NCM523; using a hydraulic press to hold the pressure for 5 min at 20–25 MPa, then dry electrodes were produced. The process is simple, scalable, solvent-free, and binder-free; the entire process from powder mixing to electrode formation can be completed in several minutes.

**Figure 10:**
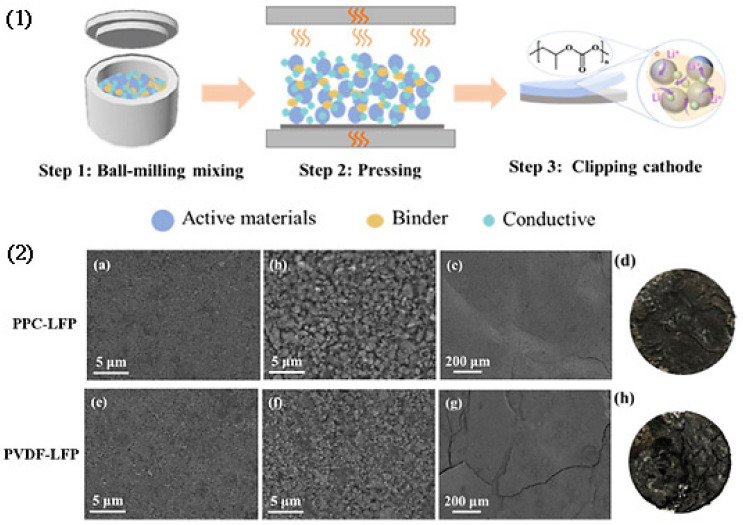
(**1**) Solvent-free dry cathode preparation process using PPC as binder [64]; (**2**) SEM images of PPC-LFP cathode (**a**) before cycle, (**b**,**c**) after cycles, and (**d**) photograph of PPC-LFP cathode after cycles. SEM images of PVDF-LFP cathode (**e**) before cycle,(**f**,**g**) after cycles, and (**h**) photograph of PVDF-LFP cathode after cycles.

The powder compression method also allows for the addition of a binder to create a conventional electrode system containing active materials, conductive agent, and binder. In 2023, Zhe Zhang et al. [64] reported a dry LFP cathode preparation process using PPC as a binder. A powder mixture of LFP, multiwall CNTs mixed with CB, and PPC with a mass ratio of 88:10:2 was first directly ball-milled for 12 h, and then hot-pressed at 120 °C to form electrodes (Figure 10a). The lithium foil was used as the anode to assemble the half-cell, and the test showed that the cathode had excellent cycling performance (capacity retention of 79.1% after 800 cycles); the electrode structure remained more intact after cycling compared with that of the battery with PVDF as the binder, thus providing a new idea for the dry electrode of the powder compression method.

### 2.5. Polymer Fibrillation

In 2006, Maxwell Technology Inc. took the lead in developing polymer fibrillation-based electrode manufacturing technology and applied for relevant patents [65,66]. This technology is mainly applied to the preparation and production of supercapacitor electrodes. In 2019, Tesla took the lead in putting this technology into the research and commercial production of lithium battery electrodes for dry processing. In recent years, this technology has become a hotspot in dry electrode technology that has been widely researched and applied [28,67,68].

Although large-scale commercial production has been achieved, the technology is very demanding in terms of the polymer binders. Polymer binders with the ability to be fibrillated are required; such binders can form a filamentary fiber network structure under the action of shear force, which is able to uniformly and firmly connect and encapsulate the electrode active particles with conductive additives and other materials to form a stable electrode active layer with minimal usage. Although some new binders have been applied to the polymer fibrillation method [69] for several years, the binder with the best practical results and the most applications is still polytetrafluoroethylene (PTFE) and its derivative materials.

**Figure 11:**
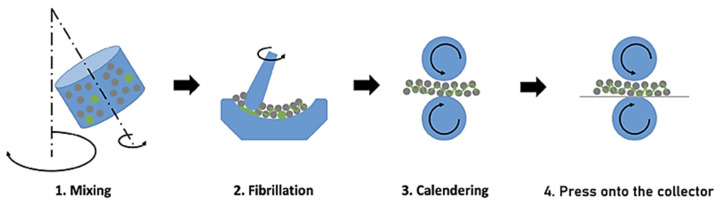
Steps of the PTFE fibrillation method electrode [73].

In the molecular chain structure of PTFE, fluorine and carbon main chain through the high bonding energy of the C-F bond are closely linked (485 kJ/mol) [70] and around the C-C main chain form a layer of low surface energy of the dense protective layer, which gives PTFE high thermal stability, solvent resistance, low coefficient of friction, and many other excellent characteristics. In addition, PTFE fine powders with a high degree of crystallinity begin to become soft above the critical transition temperature of 19 °C, at which point the friction between the fine powder particles triggers fibrillation once a shear force is applied to them. The fibers obtained can constrain the particles of the active material together and are further hot pressed or rolled to form a self-supporting electrode film. As shown in Figure 11, there are four main steps in the manufacture of dry electrodes using the PTFE fibrillation method: (1) Dry mixing of the electrode material; (2) PTFE fibrillation; the current fibrillation methods mainly include mortar and pestle milling [71] and high-speed airflow impact [72]; (3) Calendering the electrode to the target thickness. This step usually adopts roller pressing; (4) Preparation of the finished electrode: the self-supporting film is combined with the collector at a certain temperature and pressure. This process is compatible with current commercial lithium-ion battery production facilities and therefore has great potential to replace the SC process.

#### 2.5.1. The Application of PTFE in Cathodes

In 2019, Hippauf et al. [72] reported all-solid-state cathodes consisting of LiNi_0.9_Co_0.05_Mn_0.05_O_2_/solid-state electrolytes (SEs)/carbon nanofibers (CNFs)/lithium-indium alloy anode, in which they chose PTFE as the binder to prepare dry electrodes. The all-solid-state cathodes were assembled using the dry solid-state electrolyte developed by another group in a previous work [74]. By fibrillation and cold pressing process, they successfully prepared cathodes with different PTFE contents (0.1 wt.%, 0.3 wt.%, 1 wt.%) and showed by EIS tests that the resistance (53 Ω) of cathodes with PTFE contents as low as 0.1 wt.% was significantly lower than that of cathodes with 1 wt.% PTFE (263 Ω). The full battery was tested with graphite as the anode and the results showed excellent cycling performance, with a capacity retention rate of 93.6% after 100 cycles (the cut off potentials are 4.0 and 2.5 V and the charge and discharge current is 0.35 and 0.7 mA cm^−2^, respectively).

**Figure 12:**
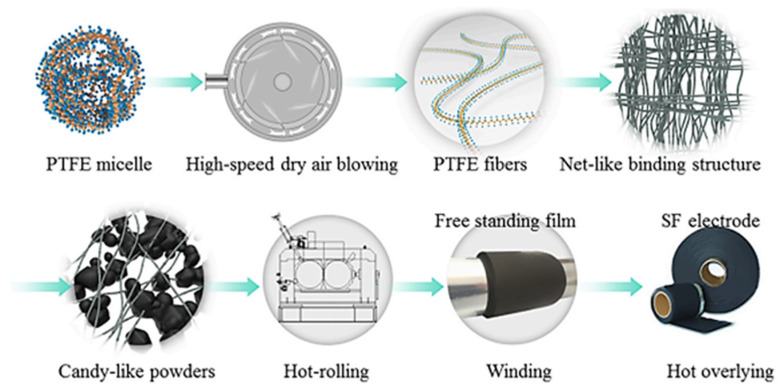
Mid-scale LFP (40% activated carbon) electrode preparation, high-speed blowing fibrillated PTFE process [75].

In 2020, Zhou et al. [75] successfully applied the polymer fibrillation dry electrode technology to the preparation of LFP electrodes (Figure 12), and PTFE was still chosen as the binder. The process combined two processes: high-speed airflow impact (for the pre-fibrillation of the mixed system of active material, conductive agent, and PTFE) and hot pressing. The thickness of the electrodes was up to 120 μm, and their compaction density was almost 1.6 times higher than that of the corresponding wet process electrodes. Super cells produced based on this method have a surface capacity of up to 1.4 mAh/cm^2^ and a volumetric energy density of 95 Wh/L, which is almost twice as high as that of wet process cells. In addition, with a self-passivating solid electrolyte interface and the formation of a stable PTFE fiber-mesh cladding structure, the assembled cell exhibits an excellent capacity retention of 92% over 5000 cycles at a high current density of 1 A/g.

**Figure 13:**
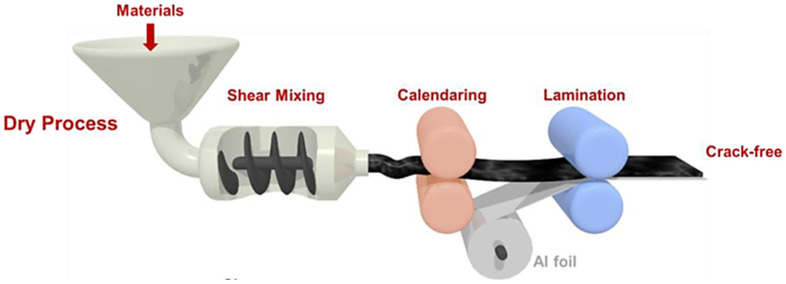
Schematic diagram of the process of preparing LiNi_0.5_Mn_1.5_O_4_ electrode based on PTFE fibrillation [76].

In addition to applications in NCM and LFP cathodes, in 2023, in the work of Weiliang Yao et al. [76] (shown in Figure 13), lithium manganese nickelate (LiNi_0.5_Mn_1.5_O_4_) electrodes were prepared with the polymer fibrillation method using PTFE as the binder. This dry electrode has ultra-high loading (~68 mg/cm^2^,~240 μm) and excellent cycling stability; assembling it (LNMO cathode) with graphite anode to form a full cell, the full cell was able to exhibit 67% capacity retention after 300 cycles at 0.1C.

#### 2.5.2. The Application of PTFE in Anodes

The polymer fibrillation method can be applied not only to the preparation of cathodes, but also to the preparation of anodes for batteries. Maxwell not only used polymer fibrillation to prepare NCM cathodes, but also used the method to prepare graphite anodes [77]. The NCM/graphite battery showed high rate performance and good cycle life under high quality loading (36 mg/cm^2^) [78]. However, they also found that there was a capacity loss when PTFE was used as the anode binder. Specifically, the initial coulombic efficiency (ICE) was significantly lower than the coulombic efficiency (CE) of the subsequent charging and discharging process, and this phenomenon occurred due to the fact that PTFE is prone to gain electrons in the negative environment, which leads to some side reactions. This problem has been reported in the study of SC electrodes employing PTFE as a cathode binder [79].

In 2022, Yang Zhang et al. [80] successfully applied PTFE fibrillation technology to graphite anodes by combining PVDF and PTFE as a composite binder. The mass ratio of each component of the electrode was graphite/carbon black/PVDF/PTFE = 90/5/2/3. The purpose of adding PVDF is to use the bonding ability of PVDF to compensate for the loss of PTFE during the first lithiation process and to continue to act as a binder to ensure the structural stability of the electrode. A full cell was assembled with the resulting electrode as the anode and the commercial LFP electrode as the cathode, and the capacity retention rate of the full cell reached more than 95% after 50 cycles. However, the problem of capacity loss in the first cycle still existed, and the ICE was 85.6%, while the CE in the second cycle increased to 99%.

**Figure 14:**
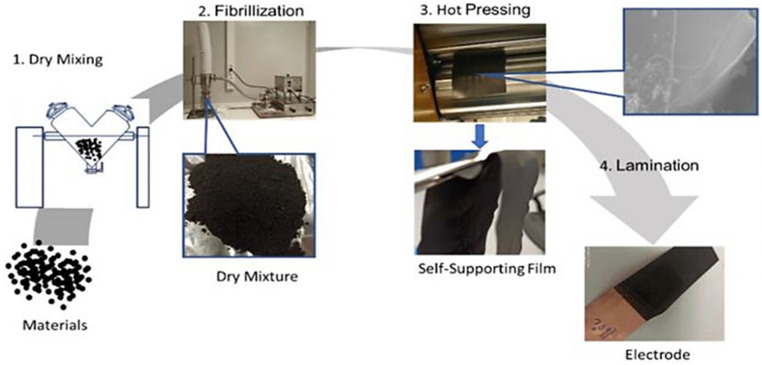
Cathodes and anodes as well as electrolyte film prepared by PTFE fibrillation process [81].

In the same year, Yang Zhang et al. [81] selected different carbon materials (graphite/soft carbon/hard carbon) as the active material, conductive carbon black as the conductive agent, and PTFE as the binder in a mass ratio of 90:5:5 to successfully prepare different dry anodes (Figure 14). Firstly, a V-mixer was used to dry-mix the electrode materials for 10 min, then a spray mill was used to fibrillate the PTFE, which was then rolled to form a self-supporting film, and finally pressed onto the carbon-coated Cu collector. The full cell was assembled using a commercial NCM523 electrode as the cathode, and the test results showed that, although the ICE was still low, the full cell with a hard carbon dry-mixed electrode as the anode exhibited excellent cycling performance (capacity retention of 90% after 120 cycles) and rate performance, which has a great potential to be applied to the Li-ion battery production line.

**Figure 15:**
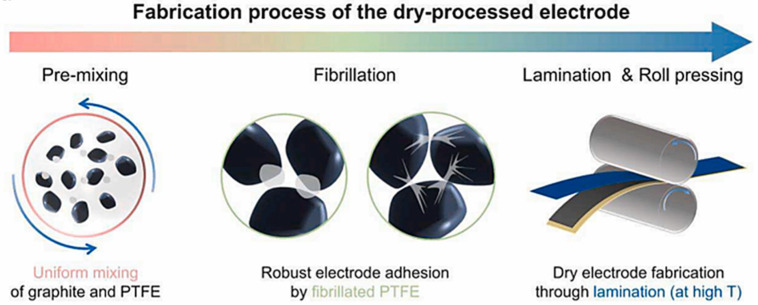
Schematic diagram of graphite anode preparation by PTFE fibrillation method [71].

In 2023, Yuri Suh et al. [71] chose graphite as the anode material and PTFE as the binder, and fibrillated PTFE using mortar and pestle milling to prepare a dry graphite anode (shown in Figure 15). The mass ratio of active material to binder was 98:2, and no conductive agent was added to the dry mix, but a conductive layer was added to the Cu collector. The test results showed that the charge transfer resistance of the dry electrode was lower than that of the corresponding SC electrode. The full cell was assembled with a commercial NCM electrode as the cathode and graphite as the anode, and the full cell achieved 88% capacity retention after 300 cycles at a current rate of 0.5C.

**Figure 16:**
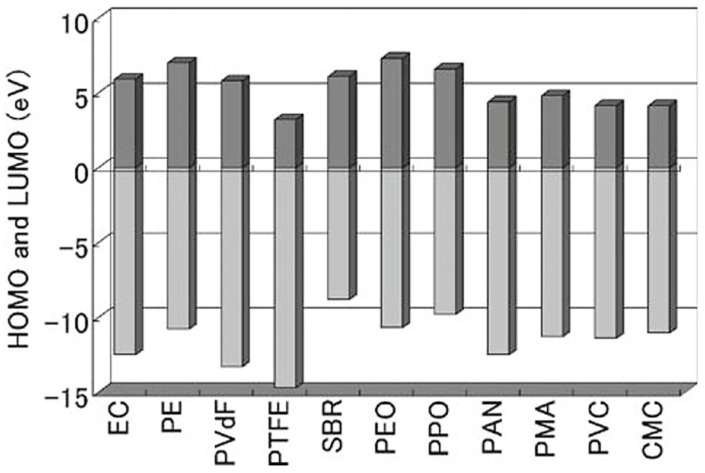
HOMO and LUMO energy levels for various Li-ion battery components [82].

The main problem in anode preparation is the instability of PTFE in anodes [79,81]. In 2009, Aisaku Nagaiwe et al. [82] used a theoretical computational model developed by them to simulate and measure the HOMO and LUMO energy levels of a number of common polymer binders, which provides a valuable reference for the selection of a binder in practical applications. A set of calculations for various polymers and ethylene carbonate (EC, a typical solvent) is given in Figure 16: each bar represents the calculated electrochemical redox window for a particular polymer. The bottom of the bar represents the HOMO energy level and its top represents the LUMO energy level. The HOMO energy levels of PTFE, PVDF, and polyacrylonitrile (PAN) are lower than those of several other polymers, a result which indicates that PTFE, PVDF, and PAN are very strong in gaining electrons and do not easily lose electrons, implying that polymers, represented by PTFE, are very stable as binders in anodes. On the other hand, after a comparison from the perspective of the LUMO energy level, it can be found that both polyethylene oxide (PEO) and polypropylene oxide (PPO) have a high LUMO energy level, among which PEO has the highest LUMO energy level, which indicates that their electron-gaining ability is very strong, and they are very prone to losing electrons, so that both PEO and PPO can be stable in the anode environment. As both of the polymers have very high HOMO, they may be unstable in positive environments. In addition, PEO and PPO are soluble in organic solvents and cannot be used as binders. Polyethylene (PE) has a high LUMO, which we would expect to be stable in negative environments, but again, it is difficult to dissolve PE in organic solvents. PVDF and SBR have almost the same LUMO energy level as PE, so these compounds can be used as anode binders. PTFE also has the lowest LUMO energy level, which means that compared to the other materials, PTFE is more prone to obtain electrons in negative environment.

**Figure 17:**
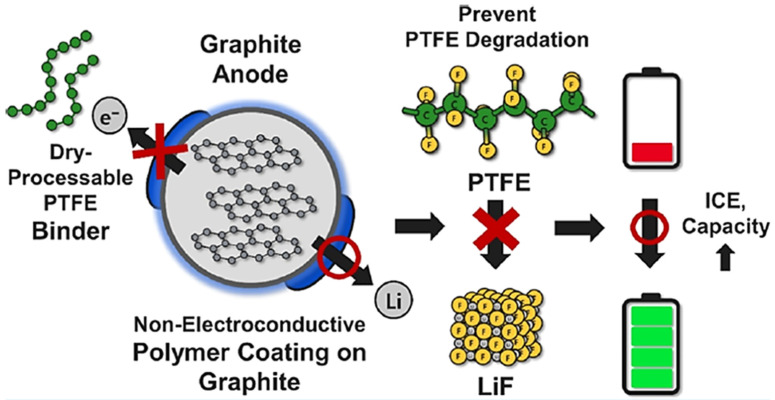
Graphite surface-coated PEO and P(VDF-TrFE-CFE) to block PTFE side reactions [83].

To solve this problem, a strategy was proposed and experimentally verified by Taegeun Lee et al. [83] in 2024, in which the graphite particles were coated with a layer of PEO or poly(vinylidene-fluoride−trifluoroethylene−chlorofluoroethylene) (P(VDF-TrFE-CFE)) that does not conduct electrons but conducts ions to partially block the occurrence of side reactions of PTFE at the anode (Figure 17). A full cell assembled with a commercial NCM electrode as the cathode was tested, and the results showed that the PEO and P(VDF-TrFE-CFE) layers increased the initial discharge capacity of the battery from 157.7 mAh/g (uncoated graphite anode) to 185.1 mAh/g and 182.5 mAh/g, respectively, and the ICE was improved from 67.2% (uncoated graphite anode) to 79.1% and 77.8%; this work provides a reliable idea for solving the instability problem of PTFE in anode.

**Figure 18:**
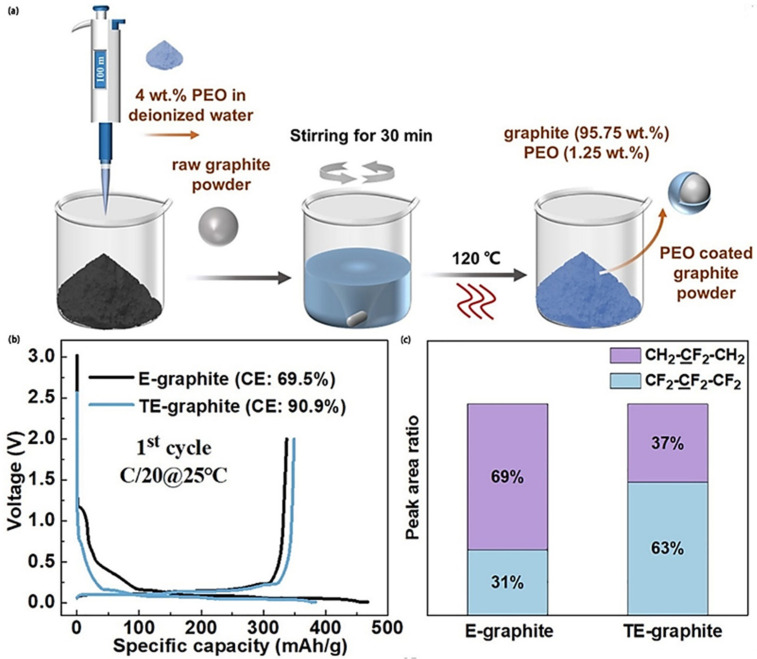
Significantly improved CE of PEO-coated graphite anode: (**a**) preparation process of PEO-coated graphite anode; (**b**) first charge/discharge curves of PEO-coated graphite anode/Li half-cell versus graphite anode/Li half-cell; (**c**) comparison of the peak intensity between graphite anode and PEO-coated graphite anode in the XPS C 1s spectra for CH_2_-CF_2_-CH_2_ and CF_2_-CF_2_-CF_2_ after cycling [84].

In the same year, Ziqi Wei et al. [84] prepared a dry graphite anode using PEO-coated graphite with PTFE as the fibrillated binder, and compared the contents of the side reaction components between the uncoated graphite anode and PEO-coated graphite anode with an XPS test, which proved that the PEO-coated graphite anode had fewer side reactions; when the half-cell was assembled using a lithium foil as the counter electrode for the test, the PEO-coated graphite anode showed a much higher CE of 90.9% than that of the uncoated graphite anode (69.5%), which again verified the feasibility of this strategy (Figure 18).

#### 2.5.3. Other Binders with the Ability to Be Fibrillated

In addition to PTFE, polymeric fibrillated binders include certain natural polymeric materials such as sericin. Similar to PTFE, sericin also forms fibers under the shear force. One possible explanation for the mechanism of fibrillation of sericin has been given by the Japanese scientist Jun Magoshi [85]. In his study, he concluded that under the action of shear force, the molecular chain of sericin unfolds and undergoes a transition from α-helix to β-folding, and at the same time the conformational transition generates oriented microfibers under a rapidly increasing shear rate, which aggregate to form fibers. The fibers formed by sericin are shorter and finer than those formed by PTFE. The smaller size of sericin fibers may be explained by the low molecular weight of this natural polymer.

In 2022, Florian Schmidt et al. [69] for the first time, applied sericin as a fibrillated binder in the manufacturing process of dry sulfur–carbon cathodes. Half-cell test results showed that PTFE-based cathodes and sericin-based cathodes exhibited initial discharge capacities of 1205 and 1203 mAh/g, respectively. After 50 cycles at 0.1C, PTFE-based cathodes delivered 633 mAh/g, whereas the sericin-based one achieved 616 mAh/g; the average CEs are 98.6% and 98.3%, respectively. Therefore, the application of sericin neither significantly improved nor significantly degraded the performance of the battery. Therefore, sericin is a promising biodegradable fluorine-free material that is environmentally friendly and is expected to be an alternative to PTFE.

Polymer fibrillation technology can be applied in both cathodes and anodes, with good scalability and compatibility, and has begun to be adopted on a large-scale commercial scale, which is very promising for replacing the SC electrode technology. However, the PTFE fibrillation method still has a significant amount to explore in terms of processing and anode stability.

Different dry processes have different application scenarios and advantages and disadvantages, as shown in Table 3; however, considering that the mainstream direction of today’s commercial Li-ion battery production line is a roll-to-roll production line, the polymer fibrillation and dry spraying deposition processes are more compatible with it. For the polymer fibrillation process, PTFE has been chosen as the binder in the mainstream direction so far; however, due to the instability of PTFE in anodes, there is a need to explore polymers with a wide electrochemical window that can be fibrillated to replace PTFE. Dry spraying deposition is suitable for both cathodes and anodes, where PVDF is mostly used. Melt extrusion and 3D printing both obtain electrode semi-finished products by means of an extruder; melt extrusion usually requires the use of a “sacrificial” binder represented by PPC; however, PVDF and PLA are mostly chosen as the binder for 3D printing. Powder compression is a simple process, suitable for the rapid production of electrodes, with or without the addition of a binder.

## 3. Advantages of the Dry Process

As an emerging process, compared with the conventional SC electrode process, the dry process has the following four main advantages: improvements in cell performance, reduction in production costs, protection of environmental resources, and broadening the range of applications.

### 3.1. Improve Cell Performance

The performance of lithium-ion batteries depends greatly on the composition and microstructure of the electrodes. Unlike SC electrodes, dry electrodes can improve area capacity and other electrochemical properties by changing the microstructure and morphology. The electrode performance achieved with the dry process is more superior, mainly in the following aspects.

#### 3.1.1. Higher Compaction Density

Increasing the compaction density of the electrodes increases the volumetric energy density of a lithium-ion battery because a high compaction density lithium-ion battery means it can be loaded with more active material for the same volume.

High volumetric energy density is important for volume-constrained application scenarios such as electric vehicles and consumer electronics. In the SC process, solvent evaporation leaves a large number of pores inside the electrode and low compaction density after calendering. The electrode porosity can be calculated with the following equation: [88].

ε = 1 − m_areal_/L (ω_AM_/ρ_AM_ + ω_B_/ρ_B_ + ω_CA_/ρ_CA_)

where m_areal_, L, ρ, and ω are surface mass load, electrode film thickness, electrode component density, and mass fraction, respectively; and the subscripts AM, B, and CA are active material, binder, and carbon additive, respectively. For commercial Li-ion batteries, the porosity of the electrodes is usually in the range of 30–45% [89]. High porosity reduces the volumetric energy density of Li-ion batteries. The compaction density achievable by the dry process can be much higher than the wet process because of the lower porosity inside the electrode. For example, the compaction density of a dry electrode with LFP as the active material is almost 1.6 times higher than the corresponding SC electrode [75].

#### 3.1.2. Better Rate Performance

**Figure 19:**
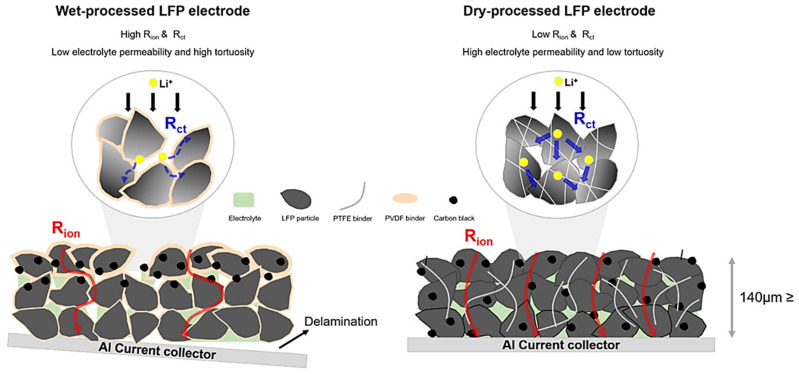
The distribution of conductive additives and polymer binder in an SC LFP electrode and a dry LFP electrode [90].

The dry process electrode has better rate performance than the wet process electrode. As shown in Figure 19 [90], a schematic diagram of the distribution of the conductive agent and binder on the surface of the active material of the SC LFP electrode and the LFP electrode obtained by the PTFE fibrillation method is given. In the dry process, the binder, the active material, and the conductive additives undergo solvent-free mixing, and the absence of an insulating layer around the active material is conducive to the transmission of electrons and ions, resulting in a better rate performance [91]. In the wet process of preparing the electrode, the binder is easy to form a tight insulating layer around the active particles, which is unfavorable for the transmission of electrons and ions, leading to a decrease in the rate performance [92].

#### 3.1.3. Larger Area Capacity

For the conventional wet process, the binder gradient distribution due to solvent evaporation and poor rate performance limits the thickness of SC electrodes [93] typically to less than 200 μm. However, if the binder and the active material are dry mixed, the binder can be uniformly distributed around the active particles. Effective ion transport contributes to the fabrication of electrodes with a high area capacity. The LFP electrodes prepared by melt extrusion can reach a thickness of 500 μm with an area capacity of 13.7 mAh/cm^2^, which is much higher than that of the electrodes prepared by the SC method [50].

#### 3.1.4. Better Mechanical Properties

Mechanical properties are important for the cycle performance of the battery, since the loss of contact between the body of the electrode material and the fluid collector is one of the most common causes of capacity loss in lithium-ion batteries. The mechanical strength of LCO cathodes [32] prepared by a dry spraying process using 5% PVDF can reach 148.8 kPa, whereas the mechanical strength of electrodes prepared by a wet process at the same ratio is only 84.3 kPa. In addition, it has been shown that dry LCO cathodes with 1% PVDF can also exhibit a mechanical strength of 93.8 kPa, which is higher than that of the corresponding wet process electrodes (83.4 kPa) [33]. The mechanical strength of LCO cathodes is also higher than that of the corresponding wet process electrodes (83.4 kPa). Improved mechanical strength also helps to avoid structural damage to the electrode during operation, which contributes to higher yields and improved safety.

For the dry process, the binder is dry mixed with the active material and uniformly distributed around the active material. The uniformly distributed binder increases the contact between the collector and the electrode film, as well as the contact between the active particles. In addition to increasing mechanical strength, the uniform binder distribution can reduce the use of binder.

As a result, the dry process can achieve higher electrode bond strength as well as higher electrode mechanical strength, compared to the wet process.

#### 3.1.5. More Ion Channels

**Figure 20:**
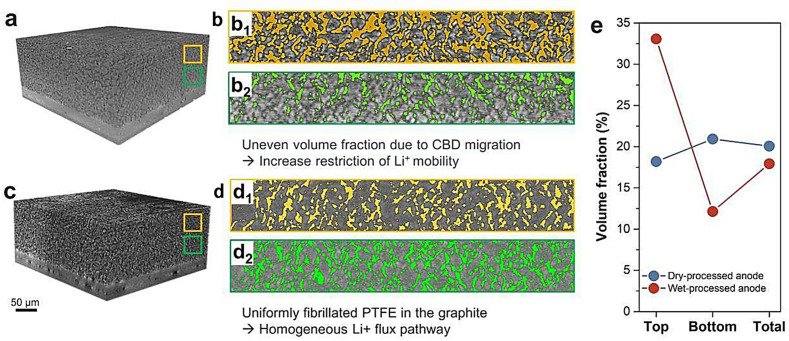
(**a**,**c**) The 3D XRM reconstructed images of SC electrode (**a**) and dry-processed electrode (**c**); (**d**) cross-sectional images of pore distribution in the top and bottom regions of wet-processed electrodes (**b1**,**b2**) and dry-processed electrodes (**d1**,**d2**); (**e**) porosity of the top, bottom, and overall layers of the wet- and dry-processed electrodes [71].

In the wet process, the binder is dissolved in the solvent and floats upwards with the solvent during drying, especially at high temperatures. The rapid evaporation of the solvent leads to the inhomogeneous distribution of the electrode material. In the work of Yuri Suh et al. [71], 3D XRM-reconstructed images of an SC graphite anode (graphite: SBR: CMC = 97:1.5:1.5) and a dry graphite anode (PTFE as the binder) as well as the porosity distributions at different locations were given, as shown in Figure 20, which shows that the dry graphite anode has a more homogeneous distribution of porosities compared with the SC electrode, which means that the dry graphite anode has a smoother lithium ion transport path, conducive to improving the battery performance.

#### 3.1.6. Fewer Residues

In the wet process, solvents and other processing aids used in the mixing process cannot be completely removed and become residues. The residual solvent in the electrode will also has side reactions with the electrolyte, resulting in the degradation of the electrode’s performance, such as reduced capacity, generation of gas, and shortened life [94,95,96]. The dry process largely avoids the residues imposed during the manufacturing process, allowing for better battery performance.

### 3.2. Reduce Production Costs

The dry process can potentially reduce costs in two ways, as follows.

#### 3.2.1. Energy Savings

For most Li-ion battery production lines, energy consumption accounts for more than 47% of the electrode manufacturing cost [97]. For example, 10 kWh of electrical energy is required to recover one kilogram of NMP [22]. 

Typically, the slurry preparation process takes several hours, and the mixing and coating drying as well as the NMP recycling processes are energy-intensive and expensive, provided that sufficient energy input is ensured [98,99,100]. In the dry process, the dry mixing process can be as short as a few minutes, depending on the choice of mixer. More importantly, the dry process does not involve drying and the recovery of solvents such as NMP [32,72]. Significant energy savings can also be achieved.

#### 3.2.2. Low Cost of Materials and Equipment

In addition to the energy savings mentioned above, these include lower raw material usage and lower investment in equipment for the dry process. For the wet process, the capital investment is higher as it requires a large plant facility to house the coater and NMP recovery system. Coaters are expensive, and the cost of NMP recovery systems is also high, with the cost of a GWh volume production line typically ranging from USD three million to USD six million [18]. The total cost of electrode production is expected to decrease by 10–15 per cent with the dry process. As shown in Table 4, a comparison [32] of the various overheads of the traditional wet process and the dry process for two lithium-ion battery production lines shows that the capital consumption of the dry process is significantly lower than that of the traditional wet process in all aspects.

### 3.3. Protection of Environmental Resources

As an example, a 10 kWh battery production line emits about 1000 kg of CO_2_ during the coating and drying process [19]. The demand for Li-ion batteries for electric vehicles is expected to exceed 1 TWh in 2030, and it is expected that 100 million tons of CO_2_ emissions would be reduced if all batteries were produced in a dry process, eliminating the coating and drying process. Another environmental issue with lithium-ion battery production is potential NMP leakage. A complex and expensive recycling system can achieve 99% recovery of NMP [22] but the remaining waste still poses a threat to the environment. NMP leakage poses a high health risk to operators and can lead to explosions when the concentration of NMP vapor is too high.

### 3.4. Broadening the Range of Applications

Due to the solvent-free nature of the dry process, it can be applied to many liquid-sensitive systems with a wider range of potential applications. Traditional liquid electrolytes face the safety issue of being flammable and explosive, and different solid electrolytes, especially sulfur-containing electrolytes, are sensitive to polar solvents, which are prone to decomposition, leading to a short cycle life. Dry electrolytes can effectively solve this problem, providing technical support for the preparation of dry electrolytes and the development of all-solid-state batteries. Secondly, the dry process can be applied to pre-lithiation, in which Li_x_Si nanoparticles are used as the pre-lithiation reagents (PreLi). However, Li_x_Si is unstable in common polar solvents (e.g., water for commercial electrode production). In addition, the complexity of slurry preparation may lead to a loss of Li_x_Si capacity, resulting in low pre-lithiation efficiency [101,102]. Therefore, the use of a dry process would eliminate the need for slurry preparation and enable efficient pre-lithiation (PreLi) with Li_x_Si nanoparticles. Another common PreLi reagent is stabilized lithium metal powder, which can only be used in non-polar solvents such as toluene [103,104]. The dry process does not use solvents, and PreLi can be directly dry-mixed with active and conductive additives. Finally, the dry electrode process is more compatible with current mainstream roll-to-roll production lines, greatly expanding its practical production applications

## 4. Challenges of the Dry Electrode Technology

With the development of electric vehicles and the growing demand for energy storage systems, the ideal dry technology battery is expected to have a high energy density and excellent cycling performance. From the production cost perspective, dry electrode technology should reduce cost and improve efficiency. At the same time, safety issues need to be taken into account. As one of the highly promising electrode manufacturing technologies, the dry process technology is expected to replace the wet process currently used on a large scale in state-of-the-art commercial lithium-ion batteries. However, a number of challenges remain before this new technology can be commercialized.

### 4.1. Study of Dry Mixing Systems

In general, dry-mixed electrode materials of different sizes and densities have a tendency to agglomerate, and in the polymer fibrillation method PTFE higher than 19 °C will produce a crystalline change [105], which is prone to agglomerate. After agglomeration, the pores between the active material and the conductive agent and binder become fewer, which is not favorable to the conductivity of the electrode. Interfacial interactions between the active material, binder, and conductive additive are critical for the uniform distribution of the dry mixture. The conductivity of the binder is generally poor, and ideally, the binder is modified on the active material with a limited degree of agglomeration to achieve the goals of low binder dosage and the high mechanical strength of the electrodes; conductive additives are also needed to improve the conductivity of the electrodes, and the conductive additives should not be allowed to agglomerate. Therefore, one of the key issues of the electrode structure design is also how to build a good conductive network. We need to adjust the amount of conductive additives and binders according to different systems. In order to optimize the dry process, the effects of surface energy and particle size on the performance of dry blends need to be further investigated, but there are relatively few related research works. In addition, the research work on the application of dry electrodes in actual battery production is insufficient, and many details need to be optimized.

### 4.2. Determination of Electrode Parameters

The thickness, compaction density, and porosity of the electrode are parameters that determine the performance of the electrode. Different dry processes have different effects on electrode parameters. Thickness, as the name implies, refers to the thickness of the final electrode after the electrode material undergoes roll pressing; generally speaking, the greater the thickness, the greater the capacity of the electrode. The thickness of dry electrodes is usually in the range of 80–150 μm; if the thickness is too low, the loading of active material will be insufficient, resulting in low capacity; if the thickness is too high, the ion transport performance may be affected. Compaction density is another important parameter, which is inversely proportional to porosity within a certain range. Like thickness, generally speaking, the higher the compaction density, the higher the capacity of the battery. However, if the compaction density is too large, the porosity is too low, many particles tend to agglomerate, the point contact is reduced, the surface contact is dominant, and the electrolyte is insufficient to infiltrate; the electronic and ionic conduction is blocked which leads to the decrease in the battery’s fast charging performance; however, if the compaction density is too small, the porosity increases, and the particles tend to form an independent entity, and the contact between them is insufficient, and it is easy to isolate the electronic conduction. Therefore, a suitable range of compaction densities can ensure that the particles are in full contact with each other without blocking the ionic movement channel, and at the same time ensuring that the electrons have good conductivity and fast ionic movement during the high-current discharge, reducing the discharge polarization, and improving the discharge platform voltage. Therefore, it is very important to determine the appropriate electrode parameters according to different processes.

### 4.3. Selection and Optimization of the Binder

Different dry processes require different binders. Fluorine-containing binders such as PVDF and PTFE have been chosen by many researchers. PVDF has a wider range of applications and is more commonly used in the dry spraying deposition method, whereas thermoplastics are mostly used in 3D printing and melt extrusion processes. So far, the dominant binder choice for the preparation of dry electrodes using the means of polymer fibrillation is still PTFE. However, in anode applications, its use is limited because it is unstable under low voltage conditions. Moreover, for cathode materials such as LFP, PTFE is not suitable for the fabrication of LFP cathodes because the hardness of LFP is too high and it is easy to break during the production and processing of electrode sheets. In order to solve the above problems, it is necessary to either explore binders that have good stability under large voltage windows and can be fibrillated or to modify existing binders such as PTFE; improvements can also be made by adding additives. Binders for other dry processes such as 3D printing and melt extrusion need to be heated above the melting point to increase the contact area. The development of suitable low melting point polymer binders is beneficial for these processes. The powder compression method mostly uses its own binder with strong bonding properties, such as PVDF, or does not add a binder but uses other materials instead.

## 5. Conclusions

The introduction of dry electrode technology in the lithium-ion battery industry has altered the microstructure and production procedures of the electrodes. In comparison to the conventional wet process, this method can maintain a sufficiently smooth ion transport pathway while preserving a large enough mechanical strength of the electrode. Dry electrode technology is a promising electrode processing technology, which mainly includes dry spraying deposition, melt extrusion, 3D printing, powder compression, and polymer fibrillation. Compared with the traditional wet electrode technology, dry electrode technology offers lower costs, higher efficiency, and significant cell performance, which has significant potential to be used in future production lines of LIBs. However, before replacing wet electrode technology, the dry electrode technology still requires further development and improvement. The dry technology still faces many challenges, such as determining the parameters of the electrode sheet and selecting appropriate binders for various dry processes. The binders used in the dry process are still under development, with the exception of PVDF and PTFE. The modification of the developed binder to meet the various requirements of the process is also a crucial topic.

## Figures and Tables

**Table 1:** Comparison of the performance of dry spraying electrodes with SC electrodes [32] (LiCO_2_:C65:PVDF = 90:5:5).

	Porosity	Bonding Strength	Capacity	Retention (0.5C, 50 Cycles)
Dry painted electrode	~30%	148.8 kPa	121 mAhg^−1^	70%
Slurry-cast electrode	~50%	84.3 kPa	<121 mAhg^−1^	58%

**Table 2:** NCA and graphite electrode composition before and after removal of “sacrificial” binder [46].

	Active Material (wt.%)	Carbon Black (wt.%)	Carbon Nanofiber (wt.%)	Binder (wt.%)	PPC Liq (wt.%)	PPC Sol (wt.%)
NCA compound	64.0	3.9	1.9	1.3	10.1	18.8
NCA electrode	90.0	5.5	2.7	1.8	-	-
Graphite compound	61.6	2.7	0.7	2.7	21.0	11.3
Graphite electrode	91.0	4.0	1.0	4.0	-	-

**Table 3:** Summary of the dry process.

Procedure	Binders	Operating Temperature	Advantages	Disadvantages	Refs.
Dry Spraying Deposition	Mostly PVDF.	20–120 °C	Can be used in almost all types of particle-based fabrications.	Hard to control mass loading, thickness, and homogeneity.	[30,31,32,33,34,35,36,37,38,39]
Hot Melting and Extrusion	PP, PW, SA, etc.	>300 °C	The process is simple and the equipment is inexpensive.	A large amount of polymer is required; high operating temperature.	[46,48,49,50]
3D printing	PVDF, PLA and other polymers.	180–230 °C	Strong designability. The shape of electrode is controllable.	Not suitable for mass production.	[57,58,86]
Powder compression	Holey graphite and PPC.	Around 120 °C	Easy to operate, and no binder is usually required.	The electrode performance is relatively poor.	[61,62,64]
Polymer Fibrillation	PTFE and sericin.	20–100 °C	Low binder content; suitable for current “roll to roll” production lines.	Unstable at anode.	[69,72,75,76,80,81,87]

**Table 4:** Cost analysis of wet and dry electrode production lines [32].

	Lithium-Ion Battery Production					
	Direct Labor (Hours/Year)	Capital Equipment (Millions)	Plant Area (Square Meters)	Direct Labor (Hours/Year)	Capital Equipment (Millions)	Plant Area (Square Meters)
Wet processing lines	511,871	109.85	12,569	595,918	139.10	15,958
Dry processing lines	441,021	94.28	10,918	499,600	112.61	13,326
Saving percentage	21.6%	14.2%	13.1%	16.2%	19.0%	16.5%

## Data Availability

Data are available in the source publications listed in the bibliography.
